# Ecological Energetic Perspectives on Responses of Nitrogen-Transforming Chemolithoautotrophic Microbiota to Changes in the Marine Environment

**DOI:** 10.3389/fmicb.2017.01246

**Published:** 2017-07-14

**Authors:** Hongyue Dang, Chen-Tung A. Chen

**Affiliations:** ^1^State Key Laboratory of Marine Environmental Science, Institute of Marine Microbes and Ecospheres, College of Ocean and Earth Sciences, Xiamen University Xiamen, China; ^2^Department of Oceanography, National Sun Yat-sen University Kaohsiung, Taiwan

**Keywords:** carbon cycle, chemolithoautotrophy, energy metabolism, global change, global warming, nitrogen cycle, ocean acidification, ocean deoxygenation

## Abstract

Transformation and mobilization of bioessential elements in the biosphere, lithosphere, atmosphere, and hydrosphere constitute the Earth’s biogeochemical cycles, which are driven mainly by microorganisms through their energy and material metabolic processes. Without microbial energy harvesting from sources of light and inorganic chemical bonds for autotrophic fixation of inorganic carbon, there would not be sustainable ecosystems in the vast ocean. Although ecological energetics (eco-energetics) has been emphasized as a core aspect of ecosystem analyses and microorganisms largely control the flow of matter and energy in marine ecosystems, marine microbial communities are rarely studied from the eco-energetic perspective. The diverse bioenergetic pathways and eco-energetic strategies of the microorganisms are essentially the outcome of biosphere-geosphere interactions over evolutionary times. The biogeochemical cycles are intimately interconnected with energy fluxes across the biosphere and the capacity of the ocean to fix inorganic carbon is generally constrained by the availability of nutrients and energy. The understanding of how microbial eco-energetic processes influence the structure and function of marine ecosystems and how they interact with the changing environment is thus fundamental to a mechanistic and predictive understanding of the marine carbon and nitrogen cycles and the trends in global change. By using major groups of chemolithoautotrophic microorganisms that participate in the marine nitrogen cycle as examples, this article examines their eco-energetic strategies, contributions to carbon cycling, and putative responses to and impacts on the various global change processes associated with global warming, ocean acidification, eutrophication, deoxygenation, and pollution. We conclude that knowledge gaps remain despite decades of tremendous research efforts. The advent of new techniques may bring the dawn to scientific breakthroughs that necessitate the multidisciplinary combination of eco-energetic, biogeochemical and “omics” studies in this field.

## Introduction

Ecological energetics (eco-energetics) is the study of energy flow and transformations in an ecosystem or through a population in a specific environment (Odum, 1968). Energy flow is a basic property of any ecosystem. From sunlit seawater to dark deep ocean and marine sediments, microorganisms employ various energy-transducing strategies to carry out ecological and biogeochemical functions (Kolber, 2007; Falkowski et al., 2008). The diverse microbial eco-energetic strategies are essentially a result of evolution during long-term biosphere-geosphere interactions (Nitschke et al., 2013; Sousa et al., 2013; Jelen et al., 2016), as vitally put by Lane et al. (2013): “if nothing in biology makes sense except in the light of evolution, nothing in evolution makes sense except in the light of energetics.”

Microorganisms constitute the most abundant, diverse and metabolically active component of biomass in the marine environment (Azam, 1998; Kallmeyer et al., 2012). The microbial communities largely control the flow of energy from abiotic forms to higher trophic levels in the ocean (Azam et al., 1983; DeLong, 2009; Brown et al., 2014). For example, marine photolithoautotrophic and chemolithoautotrophic microorganisms harvest and transform energy from otherwise largely bio-inaccessible sources (e.g., light and inorganic chemical bonds) to forms useable by chemoorganoheterotrophic consumers such as protists and animals (Brown et al., 2014). With this primary eco-energetic service, microorganisms set and control the reduction-oxidation (redox) and energy states of their environment, provide ecological services and influence the climate-mediating potential of the ocean (Azam and Malfatti, 2007; Falkowski et al., 2008; Carpenter et al., 2012). Energy is the ultimate limiting factor in determining the structure and function of the Earth ecosystem (Odum, 1968). While this makes eco-energetics “the core of ecosystem analysis” (Odum, 1968), marine microbial communities are seldomly studied from the eco-energetic perspective (Kolber, 2007; Vallino and Algar, 2016).

**FIGURE 1:**
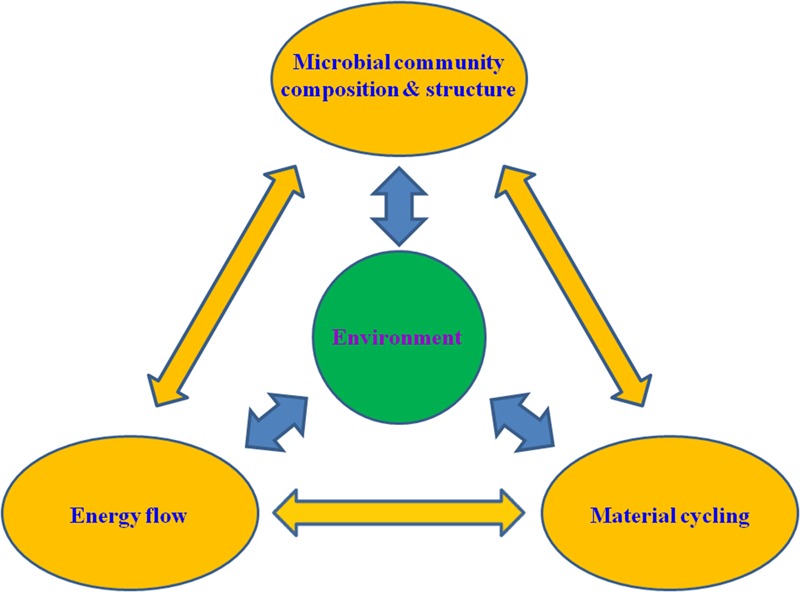
A conceptual illustration of the interactive network involving the microbial community structure, ecophysiological functions (i.e., energy flow and material cycling) and environment. The double-headed arrows indicate potential interactions between different components and processes of the network.

The marine biogeochemical cycles are driven by the microbial engines (Falkowski et al., 2008), which are mainly fuelled by energy conserved through microbial metabolic processes (**Figure 1**) (Falkowski and Godfrey, 2008; Orcutt et al., 2011). However, modeling studies of ecosystem metabolism including most recent ones usually ignored marine bacteria and archaea completely or considered them solely as decomposers (Heymans et al., 2014). This contradicts the diverse ecofunctions including the widespread autotrophy of the bacterial and archaeal communities in the ocean (Berg et al., 2010; Fuchs, 2011; Hügler and Sievert, 2011). In stark contrast to higher organisms such as plants and animals, bacteria and archaea employ diverse and complex energy metabolic pathways (Kolber, 2007), which are adapted to and effective in diverse environments. Microbial communities select energetically favorable electron donors and acceptors from their environment for energy transduction (Bar-Even et al., 2012; Eggleston et al., 2015). Even so, energy may be a limited resource for certain marine ecosystems (Burgin et al., 2011; Moore et al., 2013; Vallino and Algar, 2016). The sources and sustainability of energy supplies largely control the diversity and actual rates of the energy metabolic pathways and thus the composition, structure, and function of the microbial communities (Kolber, 2007; Dahle et al., 2015).

Microbial chemolithotrophic metabolism was discovered in the 1880s by Sergei Winogradsky, a pioneer in microbial ecology (Dworkin, 2012). Similar to photolithoautotrophs, chemolithoautotrophs contribute to primary production, which is, however, fueled by energy conserved from aerobic or anaerobic oxidation of inorganic electron donors (e.g., NH_3_, NH_4_^+^, NO_2_^-^, S^2-^, S, H_2_, CO, and Fe^2+^). Some microorganisms are chemoorganoautotrophs that oxidize organic chemicals (e.g., CH_4_) to conserve energy for carbon fixation, such as the anaerobic methane-oxidizing (ANME) archaea (Kellermann et al., 2012).

A series of exciting discoveries of new chemoautotrophic microorganisms and their bioenergetic pathways were made around the turn of the 21st century. In 1999, for example, chemolithoautotrophic anaerobic ammonium-oxidizing (anammox) bacteria were discovered and subsequently found to be widely distributed in oxygen-deficient and oxygen-depleted seawater and sediments, carrying out an important biogeochemical process in biological nitrogen removal from the ocean (Strous et al., 1999; Dalsgaard et al., 2003; Kuypers et al., 2003; Oshiki et al., 2016). The turn of the last century also witnessed the discovery of the ANME archaea and their consortial association with sulfate-reducing bacteria (Hinrichs et al., 1999; Boetius et al., 2000). ANME play an important role in the removal of methane, a potent greenhouse gas, from deep-sea cold-seep sediments and many other methane-rich environments (Knittel and Boetius, 2009; Marlow et al., 2016). Certain ANME archaea also harbor the genetic and biochemical inventory for N_2_ fixation (Pernthaler et al., 2008; Dang et al., 2009a, 2013a; Dekas et al., 2009; Miyazaki et al., 2009), thereby contributing to coupled carbon, sulfur, and nitrogen cycling in methane-rich, sulfate-rich but nitrogen-poor environments (Fulweiler, 2009). In 2002, neutrophilic, chemolithoautotrophic iron-oxidizing bacteria (FeOB) were found to be abundant in deep-sea hydrothermal vent environments (Emerson and Moyer, 2002) and they were later classified as a new class of the *Proteobacteria*: the *Zetaproteobacteria* (Emerson et al., 2007). Subsequent investigations indicate that zetaproteobacterial FeOB exist in coastal seawater and sediments as well, where they participate in coastal iron cycling and biocorrosion of man-made iron constructs (Dang et al., 2008a, 2011; McBeth et al., 2011; McBeth and Emerson, 2016). Recently, zetaproteobacterial FeOB were also discovered to utilize ferrous iron (Fe^2+^) from deep-sea basaltic rocks and basaltic glasses as an energy source, thereby contributing to carbon fixation in ultra-oligotrophic abyssal plains (Orcutt et al., 2015; Henri et al., 2016).

Since their discovery in the 1880s, chemolithoautotrophic ammonia-oxidizing bacteria (AOB) were believed to be the sole microorganisms responsible for biological ammonia oxidation in oxic environments. This long-lasting misconception was refuted in 2005 by the discovery of chemolithoautotrophic ammonia-oxidizing archaea (AOA) (Francis et al., 2005; Könneke et al., 2005), which are affiliated with the newly defined phylum, *Thaumarchaeota* (Brochier-Armanet et al., 2008; Spang et al., 2010). The most recent addition to the metabolic diversity of chemolithoautotrophic microorganisms was the bacterial strains in the nitrite-oxidizing genus *Nitrospira* capable of carrying out complete oxidation of ammonia to nitrate (comammox) (Daims et al., 2015; van Kessel et al., 2015). The history of chemoautotroph studies indicates that the ocean is full of surprises and opportunities of unknown microorganisms and novel bioenergetic strategies.

The discovery of chemolithoautotrophy by Winogradsky ended the long-lasting misconception that photoautotrophic organisms such as plants and algae are the sole primary producers on Earth (Dworkin, 2012). The discoveries of diverse chemolithic bioenergetic pathways contributed greatly to the understanding of the complexity of energy flow in Earth ecosystems and the interdependency of the biogeochemical cycles involving carbon, nitrogen, sulfur, iron, and other bioessential elements (**Table 1**). The discoveries of chemosynthetic ecosystems in the deep-sea hydrothermal vent and cold-seep environments as “oases” in the vast deep ocean “deserts” were true scientific thrills in the 70s and 80s of the 20th century, spotlighting the cornerstone species role in community structure and the primary producer role in trophic transfer the chemoautotrophic bacteria and archaea play in these sunlight-independent marine ecosystems (Felbeck and Somero, 1982; Paull et al., 1985; Jørgensen and Boetius, 2007). They provided the first evidences about the importance of microbial chemolithoautotrophy for energy and matter flows in nature and stimulated the search of life’s origin on Earth and beyond (Nisbet and Sleep, 2001; Martin et al., 2008).

**Table 1:** Typical bacterial and archaeal chemolithoautotrophic metabolic pathways found in marine environments.

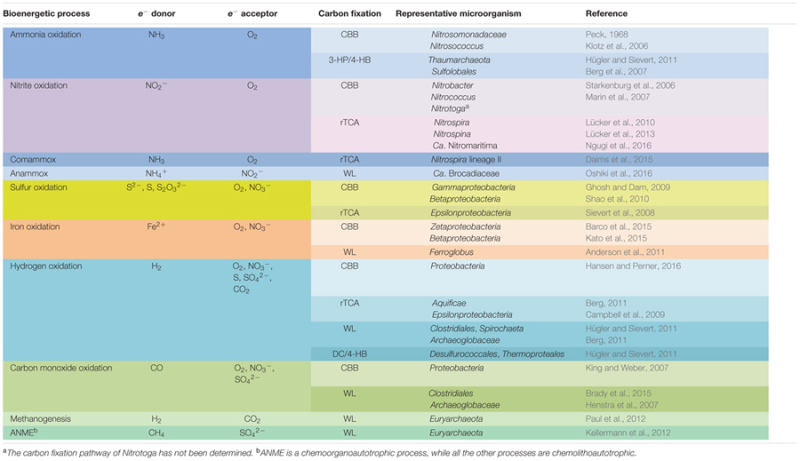

Chemolithoautotrophic microorganisms may contribute substantially to primary production in non-extreme marine environments as well. For example, dark carbon fixation in the redox transition zone of the Cariaco Basin, mainly carried out by chemolithoautotrophic sulfur-oxidizing bacteria (SOB) fueled with seawater reduced sulfur species, was equivalent to 10–333% of the local surface ocean photosynthetic primary production (Taylor et al., 2001). Microbial chemolithoautotrophs also contribute substantially to primary production in oxygenated dark oceans. Carbon fixation in meso- and bathypelagic waters of the North Atlantic, presumably by chemolithoautotrophic AOA, could amount to 15–53% of phytoplankton export production from surface water (Reinthaler et al., 2010). Dark carbon fixation in Tyrrhenian deep waters of Central Mediterranean Sea by chemolithoautotrophic microorganisms (mainly affiliated with AOA) was comparable to photosynthetic production (Yakimov et al., 2011). On the global scale, seawater AOA may fix approximately 400 Tg C y^-1^ (Hügler and Sievert, 2011). In the middle (i.e., the twilight zone) and deep ocean, chemolithoautotrophs also contribute to the production and accumulation of quite an amount (5–10 μM) of semi-labile dissolved organic carbon (DOC) (Follett et al., 2014), which may be further transformed by microorganisms to produce recalcitrant DOC (RDOC) (Jiao et al., 2014). The average turnover time of deep ocean RDOC reaches millennial timescales (Hansell, 2013). Dilution and structural recalcitrance preclude microbial consumption, constituting the major mechanisms for long-term sequestration of marine RDOC (Jiao et al., 2014; Arrieta et al., 2015; Moran et al., 2016). The deep-ocean DOC concentrations maintain small (∼40 μM) and relatively constant (Chen, 2011; Hansell, 2013), which, however, sustain active microbial communities (Arrieta et al., 2015). The *in situ* primary production of chemolithoautotrophs may provide an important source of organic matter, in addition to that released from sinking particles, hydrothermal vents and cold seeps (Chen, 2011), to fuel the activities of the deep-ocean microbiota. However, it is currently unknown whether the different sources of DOC (e.g., produced by *in situ* chemolithoautotrophs or released from sinking particles, hydrothermal vents or cold seeps) may have different molecular structures and bio-utilizabilities and thus different residence times in the ocean. Furthermore, although on average the contribution of the chemolithoautotrophic microbiota to ocean’s carbon fixation may be substantial, the *in situ* chemolithoautotrophic carbon fixation rates are highly variable among different marine environments (Taylor et al., 2001; Reinthaler et al., 2010). The *in situ* energy sources (e.g., bioavailable inorganic reductants and oxidants) may exert substantial influences on the abundance, diversity, activity, distribution, and dynamics of marine chemolithoautotrophs, and thus the energy environment may play important roles in chemolithoautotrophic carbon fixation and carbon sequestration (Dang and Jiao, 2014). Further systematic investigations are needed to quantitatively understand the roles of the microbial chemolithoautotrophs in ocean’s carbon budget and dynamics and in microbe-environment interactions.

**FIGURE 2:**
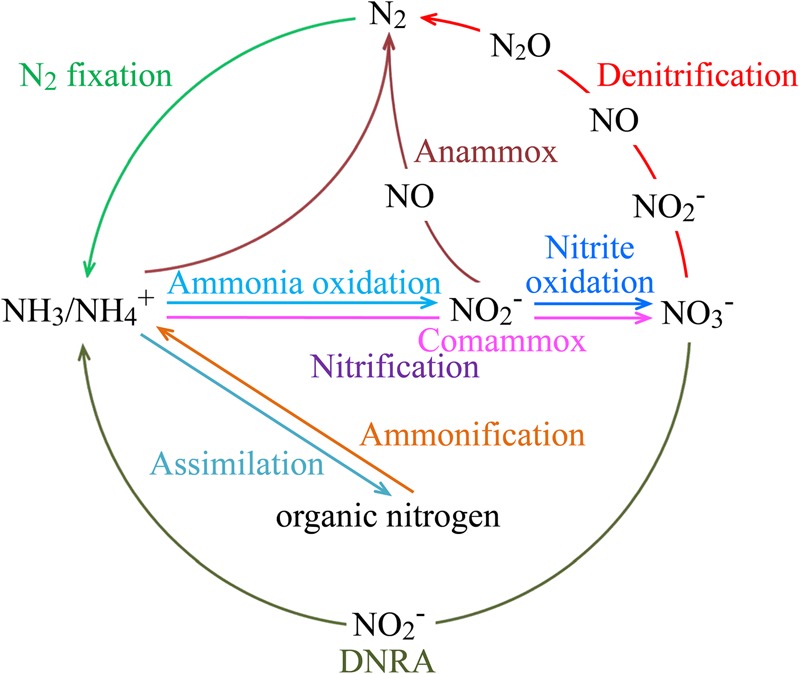
Key processes of the marine nitrogen cycle. The processes of anammox and nitrification, which includes aerobic ammonia oxidation, nitrite oxidation, and comammox, are performed by chemolithoautotrophic microorganisms. Some other nitrogen transformation processes may also involve chemoautotrophic microorganisms, such as iron-oxidizing *Zetaproteobacteria* and methanogenic and anaerobic methane-oxidizing archaea for nitrogen fixation, and *Gamma*- and *Epsilonproteobacteria* for coupled sulfur oxidation and denitrification or DNRA. Abbreviations: Anammox, anaerobic ammonium oxidation; Comammox, complete ammonia oxidation to nitrate; DNRA, dissimilatory nitrate reduction to ammonium.

Global change caused by anthropogenic activities may alter the physical, chemical and energy environment of the marine ecosystems and thus alter the spatiotemporal dynamics and functions of the microbiota (Kolber, 2007; Hutchins et al., 2009; Middelburg and Levin, 2009; Dang and Jiao, 2014). To understand how the microbial eco-energetic processes influence the structure and function of the marine ecosystems and how they respond to and exert impacts on the changing marine environment is fundamental to a mechanistic and predictive understanding of the global carbon cycle and the ocean’s climate-modulating capacity. Because of the tremendous diversity of the marine microorganisms, even just considering the chemolithoautotrophs (Berg et al., 2010; Berg, 2011; Hügler and Sievert, 2011), it is practical and meaningful to divide a community into distinct functional groups in microbial eco-energetic studies. The nitrogen cycle is probably the most perturbed biogeochemical cycle due to human activities (Rockström et al., 2009). Therefore in this review, we focus on the major functional groups of chemolithoautotrophic bacteria and archaea that are involved in marine nitrogen cycling (**Figure 2**) to tentatively illustrate their energetic strategies, ecological processes, contributions to carbon cycling, and responses to and impacts on global change associated with global warming, ocean acidification, eutrophication, deoxygenation, and pollution.

## Diverse Chemolithoautotrophic Pathways in Marine Nitrogen-Cycling Bacteria and Archaea

Dissolved inorganic nitrogen (DIN) compounds are used as either nutrients for assimilatory biomass production or electron donors or electron acceptors that are transformed by dissimilatory cellular redox reactions for microbial energy transduction (**Figure 2**). Activity of chemolithoautotrophic nitrogen-cycling bacteria and archaea is generally inhibited by light (for AOB and AOA) or by oxygen (for anammox bacteria) and outcompeted by phytoplankton for ammonium uptake in the marine photic zone (Arrigo, 2005; Merbt et al., 2012; Smith et al., 2014). Thus, DIN dissimilatory utilization for energy transduction happens mainly in the twilight and dark zones of the ocean, where aerobic oxidation of ammonia and nitrite occurs under oxic and hypoxic conditions and anammox (and denitrification) occurs under suboxic and anoxic conditions.

### Diverse Eco-energetic Strategies of Marine Chemolithoautotrophic Nitrifying Microorganisms

Nitrification is carried out mainly by chemolithoautotrophs, in two separated steps either by AOB and AOA for aerobic oxidation of ammonia to nitrite and NOB for aerobic oxidation of nitrite to nitrate or by comammox bacteria for the joint aerobic oxidation of ammonia and nitrite (Arp et al., 2007; Kuypers, 2015; Daims et al., 2016). Although most seawater environments in the surface ocean are oligotrophic, nitrification occurs throughout the water column, with the only exception likely in the core of the anoxic marine zones (Ulloa et al., 2012). Formation of the primary nitrite maximum (PNM) at the base of the marine euphotic zone in stratified water columns may be caused by phytoplankton excretion (Lomas and Lipschultz, 2006; Beman et al., 2012). However, recent studies have shown that ammonia oxidation by AOA may actually produce the major source of nitrite in PNM (Beman et al., 2013; Buchwald and Casciotti, 2013; Santoro et al., 2013). Ammonia oxidation by AOA also contributes, to variable degrees, to the formation of the secondary nitrite maximum frequently observed in the oceanic oxygen minimum zones (OMZs) (Lam et al., 2011). AOA constitute the most abundant functional group of microorganisms in the ocean’s mesopelagic and bathypelagic zones (Kirchman et al., 2007; Bristow et al., 2015), and they play a major role in nitrification and dark carbon fixation in the interior of the ocean (Herndl et al., 2005; Ingalls et al., 2006; Follett et al., 2014; Berg et al., 2015a). The deep oceans maintain a large reservoir of nitrate, associated mainly with the *in situ* AOA abundance (Herndl et al., 2005; Yakimov et al., 2011).

Ammonia-oxidizing archaea are usually more abundant and active than AOB in the ocean, particularly in oligotrophic environments (Stahl and de la Torre, 2012; Corinaldesi, 2015). The prevalence of nitrifying activity by marine AOA is mainly due to their extremely high specific affinity for ammonia and their environmental adaptivity to low concentrations of ammonium and oxygen (Martens-Habbena et al., 2009; Hatzenpichler, 2012; Stahl and de la Torre, 2012; Horak et al., 2013; Offre et al., 2014; Qin et al., 2014; Corinaldesi, 2015). Two distinct marine ecotypes (“shallow” clade vs. “deep” clade) of AOA exist (Hatzenpichler, 2012; Luo et al., 2014), each may be adapted to distinct light and nutrient regimes of the water column. Some marine AOA may utilize ammonium instead of ammonia as the preferred energy substrate (Qin et al., 2014). Many AOA can also hydrolyze urea to utilize the ureolytic products (i.e., ammonia and CO_2_) for coupled ammonia oxidation and carbon fixation (Yakimov et al., 2011; Alonso-Sáez et al., 2012; Connelly et al., 2014; Offre et al., 2014; Qin et al., 2014; Bayer et al., 2016; Tolar et al., 2016). Urea utilization thus represents a “short cut” and eco-energetically economic pathway between nitrification and carbon fixation in environmental AOA (Kirchman, 2012). Unlike AOB that use the Calvin-Benson-Bassham (CBB) cycle for carbon fixation, AOA use the most energetically efficient 3-hydroxypropionate/4-hydroxybutyrate (3-HP/4-HB) cycle pathway for carbon fixation (Hügler and Sievert, 2011; Könneke et al., 2014). AOA contribute substantially to nitrification and dark carbon fixation even in hypoxic seawater, and their activities, albeit reduced, can still be detected under sulfidic conditions (Berg et al., 2015b). Moreover, some AOA may have the capacity of coping with phosphorus scarcity in marine environments. They harbor the *pst* gene that encodes for the high-affinity, high-activity phosphate ABC transporters (Dang et al., 2013b). Some AOA also produce inorganic phosphite and organic phosphorus compounds such as phosphonates, potentially serving as phosphorus storage mechanisms for metabolic sustainability under phosphorus-starving environmental conditions (Metcalf et al., 2012; Stahl and de la Torre, 2012; Dang et al., 2013b; Van Mooy et al., 2015). The production, processing, and uptake of these phosphorus compounds (in an oxidation state of +3) are highly energetically expensive, putatively indicating the importance of phosphorus to AOA in oligotrophic environments (Dang et al., 2013b). Alternatively, these ecophysiological traits may be evolutionary relics of ancient AOA, which experienced severe scarcity of phosphorus in the pre-anthropogenic ocean (Benitez-Nelson, 2015; Van Mooy et al., 2015). Furthermore, some environmental AOA assemblages were predicted to be mixotrophic (Dang et al., 2008b, 2010c) and certain AOA isolates are able to achieve maximum bioenergetic and growth efficiency with the availability of labile organic matter (Qin et al., 2014). The AOA mixotrophic potential has been challenged by the finding that organic matter is used by certain AOA isolates for non-enzymatic detoxification of hydrogen peroxide rather than as assimilable carbon source (Kim et al., 2016). However, a recent study indicates that the genomes of certain AOA do harbor key genes that encode peroxidases and catalases for coping with oxidative stress (Sauder et al., 2017). The diverse ecotypes and ecophysiological potentials of the numerically dominant AOA warrant further systematic investigations of their contributions to the ocean’s carbon, nitrogen and phosphorus cycling and energy flux.

In marine environments, although AOA are ubiquitous and usually the dominant ammonia oxidizers, AOB may occupy particular niches and play important biogeochemical roles as well. Marine particles harbor abundant and active AOB (Karl et al., 1984). Most AOB in true marine environments are affiliated with the *Nitrosospira* genus, while *Nitrosomonas* AOB usually prevail in terrestrially impacted marine environments such as estuaries and coastal bays (Dang et al., 2010b). In stark contrast to this general AOB distribution pattern, nevertheless, marine particle-associated AOB are mainly affiliated with the *Nitrosomonas* genus (Karl et al., 1984; Phillips et al., 1999). Microbial hydrolysis of marine particle-associated organic nitrogenous compounds may produce high concentrations of NH_4_^+^ and NH_3_ (Shanks and Trent, 1979; Gotschalk and Alldredge, 1989). Locally enriched ammonia may meet the need of those AOB (e.g., certain *Nitrosomonas* bacteria) that require high ammonia availability for energy transduction. Both *Nitrosomonas* and *Nitrosospira* AOB are affiliated with *Betaproteobacteria*. In marine environments, there exists another genus of AOB, the *Nitrosococcus* that is affiliated with *Gammaproteobacteria* (Arp et al., 2007). Interestingly, *Nitrosococcus* AOB are found exclusively in marine environments and the optimal growth conditions of all known *Nitrosococcus* isolates in culture need at least 50 mM NH_4_^+^ at pH 7.5 to 8.0 (Campbell et al., 2011; Wang et al., 2016). This implies that the niche of *Nitrosococcus* AOB may be mainly associated with estuarine and coastal sedimentary environments (Wang et al., 2016), where high concentrations of NH_4_^+^ are usually found (e.g., Dang et al., 2008b, 2010b). It is also likely that the marine *Nitrosococcus* AOB are continuously under the environmental pressure of low energetic substrate availability, partially explaining their low abundance commonly found in seawater. The distinct distribution patterns of *Nitrosomonas, Nitrosospira, Nitrosococcus*, and AOA in the ocean may well reflect the different energy environments (e.g., the availability of NH_3_) they are dwelling.

To complete the nitrification process, nitrite produced by AOA and AOB needs to be further oxidized to nitrate, which is carried out by NOB. The oxidation of nitrite provides very low energy gain. Furthermore, the activity and growth of NOB may be limited by substrate availability in marine environments. These may be the major reasons that the abundance of NOB in most marine environments is usually very low (Füssel et al., 2012; Beman et al., 2013), except in the brine-seawater interface layer of the Red Sea where *Nitrospina*-like NOB may constitute up to one-third of the bacterial community (Ngugi et al., 2016). In contrast, AOA usually constitute a much higher fraction, up to or even greater than 40% of the total bacterial and archaeal community in the mesopelagic and bathypelagic zones of the ocean (Herndl et al., 2005; Kirchman et al., 2007; Bristow et al., 2015).

In spite of these bioenergetic and eco-energetic disadvantages, NOB are widespread in seawater and sediment environments. The strategy of NOB to overcome the constraint of low energy gain from nitrite oxidation is to produce high amounts of nitrite oxidoreductase, the key enzyme for nitrite oxidation (Spieck et al., 2014). This channels more cellular metabolic energy to maintenance rather than to growth. Nitrite availability has recently been identified as a key factor driving niche differentiation in NOB (Nowka et al., 2015). In addition, to overcome the problem of low and varying nitrite concentrations in oxic seawater, some NOB can degrade certain simple dissolved organic nitrogen (DON) compounds such as urea and cyanate and reciprocally feed AOB with the degradation product NH_3_ for greater nitrite production (Koch et al., 2015; Palatinszky et al., 2015). Metagenomic screening has shown that urease- and cyanase-harboring NOB may be prevalent in environments (Koch et al., 2015; Palatinszky et al., 2015). It is reasonable to hypothesize that metabolic collaborations between NOB and ammonia oxidizers (i.e., AOB and AOA) in seawater may be facilitated in marine particle- and biofilm-associated microenvironments where cross-feeding is favored (Damashek et al., 2016; Dang and Lovell, 2016).

For more than a century, nitrification was accepted as a two-step biogeochemical process carried out sequentially by ammonia oxidizers and NOB. However, it was predicted that there exist some bacteria that can catalyze comammox, a process that is energetically feasible (Costa et al., 2006). Comammox bacteria have recently been discovered as unique sublineage II *Nitrospira* (Daims et al., 2015; van Kessel et al., 2015). *Nitrospira* are globally distributed, and similarly, functional gene biomarkers of comammox bacteria have been found to be prevalent in engineered and natural environments including marine sediments (Daims et al., 2015; van Kessel et al., 2015). The combination of the divided labors of ammonia oxidizers and NOB in the comammox bacteria bestows certain eco-energetic advantages, including facilitated acquisition of nitrite as an energy substrate and enhanced energy yield in terms of adenosine triphosphate (ATP) production (Costa et al., 2006; Daims et al., 2016). However, comammox also confers certain disadvantages to the bacteria. ATP is produced for catalytic purposes rather than for energy storage in cells (Pfeiffer and Schuster, 2005). The long metabolic pathway of comammox lowers the ATP production rate and thus the maximal growth rate the bacteria can achieve (Costa et al., 2006). The comammox process is predicted to be favored when ammonia and nitrite as energetic substrates are limiting and replenished slowly and when bacteria grow in clonal patches such as in biofilms (Costa et al., 2006). So far, no comammox bacteria have been found in marine waters (Kuypers, 2015). However, particles in seawater may present unique niches for comammox bacteria.

### The Unique Eco-energetic Mechanism of Chemolithoautotrophic Anammox Bacteria

In oxygen-deficient and oxygen-depleted sediments and marine waters such as those occurring permanently in oceanic OMZs and seasonally in eutrophic coastal areas, microorganisms carrying out anammox and denitrification contribute to fixed nitrogen removal (Devol, 2015). Anammox bacteria employ the redox reaction of coupled nitrite reduction and ammonium oxidation for energy transduction to fix carbon (Kartal et al., 2013; Oshiki et al., 2016), while bacterial and archaeal denitrifiers are usually heterotrophs that use organic matter as electron donors for stepwise reduction of nitrate, nitrite, NO, and N_2_O to produce N_2_ (Carolan et al., 2015; Devol, 2015). Anammox bacteria harbor anammoxosomes, unique organelles functionally analogous to eukaryotic mitochondria to perform the energetic reaction (van Niftrik and Jetten, 2012; Jogler, 2014). Anammox bacteria are affiliated with a narrow bacterial clade, the *Candidatus* Brocadiales order of *Planctomycetes* (Kartal et al., 2013). In addition to physiological specialization and phylogenetic segregation, niche separation is also prevalent in anammox bacteria. *Ca.* Scalindua mainly occur in marine environments and all the other anammox bacterial genera are adapted to low-salinity habitats (Kartal et al., 2013). Estuaries are an exception where *Ca.* Scalindua and some freshwater anammox bacteria may both exist (Dang et al., 2010a, 2013c). In contrast, marine denitrifiers are common in *Bacteria* and *Archaea*, a phenomenon likely being facilitated by horizontal gene transfer for denitrifying trait spreading (Jones et al., 2008). Furthermore, most denitrifiers are facultative anaerobes and can rapidly switch among different energetic pathways in response to changing environmental conditions (Dang and Jiao, 2014), while anammox bacteria are obligate anaerobes and they may prefer stable environmental conditions.

As anammox bacteria and denitrifying microorganisms occupy similar environments, they may compete for nitrate and nitrite (as electron acceptors) for energy transduction. The bioavailability of organic carbon and organic matter stoichiometry may be key factors determining the relative contributions of anammox and denitrification to fixed nitrogen removal in the ocean (Thamdrup and Dalsgaard, 2002; Engström et al., 2005; Babbin et al., 2014; Chang et al., 2014; Babbin et al., 2016). Most organic compounds that are microbiologically utilizable as electron donors can be more easily oxidized and thus more energy-favorable than ammonium. This energetic difference influences the distribution of the anammox bacteria and denitrifiers in the ocean and their relative contributions to the marine nitrogen and carbon cycling (Dang et al., 2009b, 2010a; Ulloa et al., 2012). In suboxic and anoxic environments that are rich in organic matter such as some eutrophic coastal waters and sediments, heterotrophic denitrifiers usually outperform chemolithoautotrophic anammox bacteria for fixed nitrogen removal, while in certain oceanic OMZs the contribution of anammox bacteria may match or even outperform that of the heterotrophic denitrifiers (Ward et al., 2009; Ulloa et al., 2012). Similarly, organic-poor deep-sea sediments usually favor anammox activity over denitrification (Jaeschke et al., 2010; Devol, 2015). However, the influence of organic carbon on the partitioning of nitrogen loss between anammox and denitrification may be more complicated than previously thought. It was found recently that organic matter enrichment may stimulate dissimilatory nitrate reduction to ammonium (DNRA), competing against denitrification for nitrate acquisition (Brin et al., 2017). The complex microbial nitrogen transformation processes and their distinctly different responses to specific suboxic and anoxic marine environments constitute an obstacle to a simple and predictive understanding of the microbe-environment interactions.

## Eco-Energetic Responses of Nitrogen-Cycling Chemolithoautotrophs to Global Change Impacts

Chemolithoautotrophic microorganisms have been playing critical roles in shaping the Earth’s environment and planetary evolution (Fuchs, 2011; Martin et al., 2014). Although photoautotrophs (i.e., cyanobacteria, algae, and plants) are the most dominant carbon fixers on the surface layer of land and ocean, chemolithoautotrophic microorganisms have been carrying out carbon fixation long before the advent of oxygenic photosynthetic organisms (Fuchs, 2011; Braakman and Smith, 2012; Jelen et al., 2016). Some chemolithoautotrophs were probably among the first organisms on earth and have played a key role in driving the transition of earth from its primordial inorganic state to a state rich in biogenic organic matter. They may have made substantial impacts on the geochemical condition and redox status of the primitive Earth, leading to the origination and evolution of the planet ecosystems and biogeochemical cycles.

Anthropogenic activities have significantly changed the Earth’s environments and ecosystems as well (Duarte, 2014; Brondizio et al., 2016). Rapid increase in CO_2_ emission as a result of fossil fuel consumption has led to various environmental problems such as global warming, ocean acidification, and ocean hypoxia. Carbon perturbation induced by ongoing global change is further compounded by other harmful human activities such as environmental pollution and eutrophication (Bijma et al., 2013). How these anthropogenic environmental disturbances influence the eco-energetics and biogeochemical functions of the marine microbiota warrants in-depth examinations, particularly for the chemolithoautotrophic microorganisms that play important roles in carbon fixation and carbon sequestration of the ocean.

### Chemolithoautotrophic Responses to Global Warming

Temperature is an important environmental factor that influences microbial ecophysiology and biogeochemical functioning in multiple and profound ways. For example, the permeability of proton across cytoplasmic membranes, which plays a critical role in microbial bioenergetic processes, increases with environmental temperature (Tolner et al., 1997; Berry, 2002). Proton leakage lowers the microbial bioenergetic efficiency. Therefore, microorganisms continuously monitor surrounding temperature and abrupt temperature changes usually induce rapid microbial stress responses (Schumann, 2009). It was predicted that a 1°C increase of seawater temperature in the bathypelagic ocean may cause a 55% increase of heterotrophic production by the *in situ* bacterial and archaeal community (Lønborg et al., 2016). However, autotrophic and heterotrophic microorganisms may respond to ocean warming differently, leading to changes in the ocean’s metabolic balance (Lønborg et al., 2016).

Nitrogenous nutrient scarcity tends to limit primary production of the phytoplankton communities throughout much of the low-latitude surface ocean (Moore et al., 2013). Eco-energetically, chemolithoautotrophic nitrogen-cycling microorganisms carry out dissimilatory transformations of nitrogenous compounds, resulting in the compositional changes and/or loss of nitrogenous nutrients from environments (Stein and Klotz, 2016). They contribute to marine carbon cycling not only by direct carbon fixation but also by their control on the speciation, concentration, and distribution of nitrogenous nutrients in the ocean, thus influencing the composition, structure, activity, and spatiotemporal dynamics of the photosynthetic communities and their primary productivity. The interactions of nitrogen-cycling microorganisms with photosynthetic communities via the linkage of nutrients are highly complex and influenced by diverse factors. For example, the deep ocean is usually rich in nitrate, resulted mainly from the activities of chemolithoautotrophic ammonia- and nitrite-oxidizers. However, the deep ocean is devoid of sun light and thus phototrophic activities. This creates a spatial decoupling of nutrients with solar light energy, limiting substantially the primary productivity and carbon sequestration capacity of the ocean. With the impact of global warming, the surface ocean is becoming warmer and more stratified, aggravating the spatial decoupling of deep ocean nutrients and surface ocean photosynthetic CO_2_ fixation.

Aerobic ammonia oxidation by AOA has been found as one of the highest energy-yielding chemolithotrophic processes in high temperature environments such as hot springs (Dodsworth et al., 2012; Hatzenpichler, 2012). Physiological, genomic, and phylogenetic analyses suggest that the ancestor of AOA was thermophilic and a number of studies support this inference by showing the prevalence of AOA in terrestrial hot spring and coastal and deep-sea hydrothermal vent environments (Hatzenpichler, 2012; Stahl and de la Torre, 2012; Wang L. et al., 2015). The ubiquity of abundant AOA in mesopelagic and bathypelagic seawater and deep-sea sediments suggests that their mesophilic and psychrophilic physiology may be the result of secondary adaptations (Hatzenpichler, 2012; Stahl and de la Torre, 2012). On the contrary, AOB may have a mesophilic origin. A microcosm experiment showed that the composition of soil AOB usually changes very little with increasing temperature, while the abundance and ammonia oxidation potential activity of certain AOA phylotypes increase significantly with increasing temperature (Tourna et al., 2008). In line with this, a recent study showed that the biochemical processes of ammonia oxidation may be thermodynamically different between soil AOA and AOB, with AOA having a significantly higher minimum temperature than AOB for ammonia-oxidizing activity (Taylor et al., 2017). Although it is unknown if marine AOA and AOB bear a similar thermodynamic difference, temperature was putatively identified as a key environmental factor affecting the composition and distribution of the AOA assemblages in sediments of the South China Sea, a large marginal sea in the subtropical and tropical Pacific Ocean (Dang et al., 2013b). Does this mean that the marine AOA may be more responsive than AOB to ocean warming? The polar ecosystems are currently facing strong global warming effects (Schofield et al., 2010; Spielhagen et al., 2011). However, a recent investigation in western coastal Arctic seawater shows that nitrification rates, likely contributed mainly by AOA, are resistant to short-term temperature elevations (Baer et al., 2014). A common trend of the warming effects on the diverse marine nitrifying microbiota cannot be concluded yet. How and to what extent elevated seawater temperatures caused by global warming may directly affect the ecophysiology of marine AOB, AOA, and NOB are still difficult to predict.

In addition to the direct effects, global warming may also exert certain indirect effects on the marine nitrifying microbiota and their eco-energetic and carbon-fixing activities. Global warming causes ocean stratification that weakens the vertical mixing of seawater and makes surface water of the open oceans to be more oligotrophic. This effect may lower phytoplankton primary productivity and thus the export of particulate organic matter (POM) from the surface ocean to the deep ocean and sediments (Bijma et al., 2013; Turner, 2015). Marine particles and sinking phytoplankton aggregates are the hotspots of extracellular enzyme activities, contributing to microbial degradation of POM polymeric organic matter and release of nutrients and energy substrates such as ammonium, phosphate and labile DOC in the middle and deep waters of the ocean (Azam and Malfatti, 2007; Wright et al., 2012; Ploug and Bergkvist, 2015; Dang and Lovell, 2016; Krupke et al., 2016). Certain studies have suggested enhanced nitrification activities in marine waters that are rich in particles and phytoplankton aggregates (Karl et al., 1984; Klawonn et al., 2015a; Damashek et al., 2016). Diminished biological pump (BP) function caused by the global warming effects reduces not only the ocean’s carbon sequestration capacity but also the nutrient replenishment rate in the interior of the ocean. Diminished ammonium supply due to weakened BP exports may lead to reductions of the nitrifiers’ activities such as nitrate production and carbon fixation in the marine mesopelagic and bathypelagic zones.

A number of studies have shown that the anammox bacteria are well adapted to low-temperature environments such as deep-sea and polar region sediments (Rysgaard et al., 2004; Jaeschke et al., 2010; Russ et al., 2013; Canion et al., 2014a,b; Shao et al., 2014; Sonthiphand et al., 2014). Anammox bacteria can alter the composition of their cell membranes and thus enhance the membrane fluidity by increasing the content of short chain ladderane fatty acids in response to low-temperature conditions (Rattray, 2008). In cold environments, anammox bacteria are found to carry out psychrophilic anammox process, while denitrifying bacteria usually carry out psychrotrophic nitrogen removal process (Rysgaard et al., 2004; Canion et al., 2014a,b). Colder temperature thus may be an important environmental factor that favors anammox over denitrification in deep-ocean and polar sea sedimentary environments (Rysgaard et al., 2004; Canion et al., 2014a,b; Shao et al., 2014). The different temperature adaptations between anammox bacteria and denitrifiers suggest that increased temperatures caused by global warming may favor denitrification over anammox in cold marine environments. However, in a recent study, the psychrophilic physiology of anammox bacteria could not be verified and anammox and denitrification were found to have similar temperature responses, which are not influenced by warming in temperate coastal environments (Brin et al., 2017). Another study found little changes of community structure and activity rate of anammox bacteria and denitrifiers in response to increased temperatures, suggesting both microbial groups may be ecophysiologically tolerant to climate warming disturbances (Canion et al., 2014b). More systematic ecology studies covering broader environmental conditions are necessary for revealing the true ecophysiological characteristics of the marine anammox bacteria and denitrifiers.

The anammox and denitrification microbial assemblages may be influenced by global warming via its indirect effects. Although both denitrification and anammox activities may be enhanced by marine particles and phytoplankton aggregates, seawater anammox bacteria are frequently found as free-living microorganisms except in a few cases (Woebken et al., 2007; Ganesh et al., 2015; Stief et al., 2016). Dissolved organic matter (DOM) and POM usually stimulate denitrification rates by providing the necessary energy and carbon sources for the denitrifiers (Canion et al., 2014b; Chang et al., 2014; Babbin et al., 2016), while the chemolithoautotrophic anammox bacteria rely less on organic matter for energy and material metabolisms. Therefore, reductions in POM flux and DOC availability induced by global warming may affect more negatively on denitrification than on anammox in the ocean’s mesopelagic and bathypelagic zones and the deep-sea sediments (Canion et al., 2014b). However, in temperate estuarine and coastal sediment environments, a recent study showed that the role of organic matter in altering nitrogen removal partitioning between anammox and denitrification cannot be verified (Brin et al., 2017).

The controversial effects of temperature and organic matter on the activities of anammox and denitrification indicate the complexity of the microbial responses to the global warming effects. Microbial communities in different marine environments may have developed different ecophysiologies and habitat adaptivities. The key limiting environmental factors that control the anammox and denitrifying activities in different environments may be different as well. For example, microbial assemblages in temperate estuarine and coastal environments experience obvious seasonal changes in temperature, nutrients and various sources of organic matter inputs. In addition, estuarine and coastal environments usually experience eutrophication and pollutions. The microbiota here may have developed adaptations to these varying factors. Moreover, the different microbial nitrogen transforming processes may also be influenced by the scarcity of various trace metals as enzyme cofactors and/or by the toxicity of diverse heavy metals to enzymes, a critical scientific question in microbial eco-energetics that is not yet clearly solved (Klotz and Stein, 2008; Simon and Klotz, 2013; Glass et al., 2015; Löscher et al., 2016). This implies that some environmental conditions, other than temperature and organic matter, may be the most important factors controlling the denitrification and anammox activities in certain marine environments. Furthermore, complex interactions among the different microbial functional groups and between microorganisms and other organisms in the ecosystems may all influence the outcomes of the global warming effects. For example, addition of extra DOM or POM may stimulate the DNRA activity rather than the denitrification activity (Brin et al., 2017). The effects of protozoan grazing and viral lysis on the partitioning of nitrogen loss between denitrification and anammox in marine environments are not resolved, either (Löscher et al., 2016). The high degrees of complexity and uncertainty indicate that there is the need of more systematic investigations for a comprehensive and accurate understanding of the responses of marine anammox and denitrifying microorganisms to the impacts of ocean warming.

### Chemolithoautotrophic Responses to Ocean Acidification

Increased anthropogenic CO_2_ emission causes not only global warming, but also increased partial pressure of CO_2_ (*p*CO_2_) and hence decreased pH in seawater. The most significant drops of pH are usually associated with estuarine and coastal seawater, caused additionally by terrestrial, anthropogenic, mixing and upwelling inputs of nutrients and organic matter that lead to enhanced primary production and microbial respiration (Cai et al., 2011; Lui et al., 2015). Increased atmospheric deposition of nitrogen and sulfur in coastal regions resulting from fossil fuel combustion and agricultural fertilizer application also lowers seawater pH (Doney et al., 2007; Hagens et al., 2014). Acidification may influence the ocean’s primary productivity and carbon sequestration capacity (Doney et al., 2009). Acidification also changes the equilibrium of the ocean’s carbonate chemistry system, leading to stresses and damages to certain sensitive ecosystems such as the shallow coral reefs (Andersson and Gledhill, 2013; O’Brien et al., 2016). Ocean acidification may reduce marine biodiversity and fisheries as well, due to acidification-induced animal physiological stresses and/or acidification-induced changes in the ecosystem’s trophic transfer efficiency (Widdicombe and Spicer, 2008; Branch et al., 2013; Cripps et al., 2016; van Leeuwen et al., 2016).

Ocean acidification may exert significant impacts on marine biogeochemical cycles. For example, microbial photosynthesis and nitrogen fixation have been found to be enhanced under acidification conditions in the surface ocean, which may be related directly to enhanced inorganic carbon assimilation due to increased seawater *p*CO_2_ (O’Brien et al., 2016). However, ocean acidification exerts a negative impact on the chemolithoautotrophic ammonia oxidation process. Ocean acidification changes the seawater NH_3_/NH_4_^+^ equilibrium by ionizing more ammonia molecules to form ammonium cations. A 0.3 pH decrease projected to happen by the year 2100 (Caldeira and Wickett, 2005) may cause a 50% decrease of the seawater NH_3_ concentration (Zeebe and Wolf-Gladrow, 2001). Studies have shown that this shift in NH_3_/NH_4_^+^ equilibrium may directly reduce the ocean’s ammonia oxidation rate (Beman et al., 2011). Although marine sediments have certain buffering effect against porewater pH changes (Kitidis et al., 2011), decreases of benthic nitrification rate have also been reported in some investigations (Braeckman et al., 2014). However, the change of nitrite oxidation activity in response to ocean acidification may be quite different from that of ammonia oxidation activity. The response of the two-step nitrification process to ocean acidification may be more complicated that previous thought. A recent study showed that the nitrite oxidation rates of coastal seawater correlate positively with [H^+^] and thus negatively with pH (Heiss and Fulweiler, 2016). Currently it is not clear if this phenomenon is common in the world oceans, nor is known about the mechanism for this NOB response.

Furthermore, global warming may exert a compound effect along with ocean acidification on the change of marine nitrification. It was found that high temperature in summer had an inhibitory effect on the activity and growth of NOB, leading to the decoupling of ammonia oxidation and nitrite oxidation and thus the accumulation of nitrite in seawater (Bristow et al., 2015; Schaefer and Hollibaugh, 2017). Some NOB such as *Nitrotoga* spp. prefer environments with a slightly acidic pH and low-temperatures (<20°C) in physiological experiments (Hüpeden et al., 2016). Global warming increases the temperature of both the surface ocean and the ocean’s interior (Masuda et al., 2010; Mora et al., 2013; Levin and Le Bris, 2015), thus it may negatively influence the rate of nitrite oxidation in certain environments of the ocean. Under the combined influences of ocean acidification and global warming, the chemical composition of the ocean’s nitrogenous nutrient reservoir may be altered. It is well known that nitrate is the primary inorganic nitrogen source for marine diatoms, which contribute substantially to the BP-mediated particulate organic carbon (POC) export and storage in the ocean’s interior (Bowler et al., 2010; Beman et al., 2011; Diner et al., 2016). On the contrary, marine dinoflagellates were found to be favored by ammonium, which also enhances algal bloom formation and toxin production (Leong et al., 2004; Hattenrath-Lehmann et al., 2015). The lowered nitrate pool due to ocean acidification- and warming-induced decrease of nitrification may lower diatoms-mediated primary production and POC-mediated carbon sequestration of the ocean but increase the incidences of harmful algal blooms by dinoflagellates.

Although certain soil AOB and AOA strains such as *Ca.* Nitrosotalea devanaterra have been found to be obligate acidophiles (Hayatsu, 1993; Lehtovirta-Morley et al., 2016), it is doubtable that the majority of the marine AOB and AOA are acidophiles because the seawater pH is usually above 7. The long-term lack of acidic conditions makes it difficult for marine AOB and AOA to evolve genetic and physiological mechanisms to become acidophilic. However, not all the bacterial and archaeal ammonia oxidizers respond to ocean acidification in the same way. Ocean acidification may change the composition of the ammonia-oxidizing communities, in which urease-harboring AOA and AOB may gain more importance as they can use urea as a source of ammonia and CO_2_ for both energy transduction and carbon fixation (Koper et al., 2004; Klotz et al., 2006; Kirchman, 2012; Bowen et al., 2013). Urea is the most abundant chemical species of labile low-molecular-weight DON in the ocean (Solomon et al., 2010). Rapid autochthonous production by bacteria, algae, protists, and animals (e.g., zooplankton, mollusks, crustaceans, fish, and mammals) and various allochthonous inputs such as from agricultural fertilizers make urea an important constituent of the marine nitrogen cycle (Berman and Bronk, 2003; Glibert et al., 2006; Solomon et al., 2010). The urea molecule is uncharged and its chemistry does not change with pH. Urea is prevalent in seawater and sediment porewater (Glibert et al., 2006; Solomon et al., 2010), likely serving as a preferred and important nitrogen source for ureolytic ammonia oxidizers under acidified conditions (Pommerening-Röser and Koops, 2005; Lu and Jia, 2013). Indeed, ureolytic AOA have been found as the major ammonia oxidizers contributing significantly to the nitrification activity in certain marine environments (Alonso-Sáez et al., 2012; Tolar et al., 2016). Urea as an alternative substrate source for microbial ammonia oxidation may compensate, to currently unknown degrees, for the reduction of the marine nitrification activities caused by ocean acidification. In addition, some studies showed that the nitrite oxidation rates are greater than the ammonia oxidation rates in coastal seawater (Heiss and Fulweiler, 2016). Some sources of nitrite, other than that provided by *in situ* ammonia oxidation, may provide extra nitrite for the activity of NOB. Seawater cyanate may be used by NOB for “reciprocal feeding” of ammonia oxidizers to obtain extra nitrite (Palatinszky et al., 2015; Heiss and Fulweiler, 2016). However, currently the quantitative contributions of ureolytic ammonia oxidizers and cyanate-degrading NOB to nitrification in the global ocean under acidification conditions have not been in-depth and systematically investigated.

Ocean acidification may enhance anammox activity, partially resulting from increased ammonium concentrations due to the shift of the NH_3_/NH_4_^+^ equilibrium under acidification conditions (Widdicombe and Needham, 2007; Gazeau et al., 2014; Tait et al., 2014). Ocean acidification may enhance the activity of denitrification as well, because the NO_3_^-^, NO_2_^-^, and NO reductases are more active at neutral or lower pH (Richardson et al., 2009). Therefore, it is likely that fixed nitrogen loss from the ocean will increase due to ocean acidification. Furthermore, ocean acidification may cause an increase of greenhouse gas N_2_O production as N_2_O reductase is sensitive to pH and less active at pH < 7 (Richardson et al., 2009). A recent study suggests that low pH interferes with the N_2_O reductase assembly, putatively revealing a molecular mechanism of the acidification effect on N_2_O dynamics (Liu et al., 2014). Acidification may increase N_2_O production by aquatic ammonia-oxidizers, as well (Frame et al., 2017). Seawater N_2_O production is further compounded by many macroscopic processes of the ocean, such as vertical mixing and upwelling (Tseng et al., 2016). In summary, ocean acidification may cause altered (likely decreased) nitrification, increased N_2_O emission and increased loss of nitrogenous nutrients via enhanced denitrification and anammox, among many other significantly altered biogeochemical processes in the ocean. It is necessary to develop a mechanistic and quantitative understanding of the various marine nitrogen-cycling processes and the ocean acidification effects, which are fundamentally important for better modeling and predication of the behavior and function of the future marine nitrogen and carbon cycles.

### Chemolithoautotrophic Responses to Ocean Eutrophication and Deoxygenation

The trophic and oxygenation states are two critical and interconnected factors that have significant influences on the ecological processes and biogeochemical functions of the ocean’s ecosystems. Anthropogenic eutrophication and upwelling are the major contributors to coastal hypoxia and anoxia, which may be additionally enhanced by incoming offshore seawater in certain marginal seas (Lui et al., 2014). Coastal eutrophication is predicted to increase, due to continuing increase of human activities (Doney, 2010; Nogales et al., 2011; Statham, 2012; Bijma et al., 2013). Under the impact of ongoing global change, the duration and intensity of most of the large-scale upwelling systems are predicted to increase as well (Sydeman et al., 2014; Wang D. et al., 2015). Global warming exacerbates the frequency, extent and impacts of coastal “dead zones,” which usually occur during warm seasons. The oceanic OMZs will also intensify and expand due to warming-induced oxygen solubility reduction and seawater stratification (Keeling et al., 2010; Wright et al., 2012). Therefore, oceanic OMZs and coastal hypoxia and anoxia will undoubtedly exacerbate in the future. These situations may alter the major metabolic pathways and functional services of the affected ecosystems.

Hypoxic and anoxic environments may facilitate microbial nitrogen fixation (Zhou et al., 2016). Under O_2_-rich environments, bacterial nitrogen acquisition through nitrogen fixation is eco-energetically less favorable than assimilatory nitrate uptake (Eichner et al., 2014; Jiang et al., 2015). However, in hypoxic environments such as the marine OMZs, nitrogen fixation may be eco-energetically more favorable and less inhibited by high nitrate concentrations (Großkopf and Laroche, 2012). Because most of the diazotrophs in the aphotic marine hypoxic and anoxic environments are heterotrophs, the availability of metabolizable organic substrates as energy sources is an important factor influencing the abundance and activity of the *in situ* diazotrophs. Alternatively, chemolithoautotrophic ANME-2c archaea may contribute substantially to nitrogen fixation in methane-rich environments (Pernthaler et al., 2008; Dang et al., 2009a; Dekas et al., 2009; Miyazaki et al., 2009). Furthermore, the diazotrophic abundance and activity may also be controlled by the availability of phosphate and/or iron, which may vary in different marine environments (Moore et al., 2013; Dang and Lovell, 2016). Diazotrophic microorganisms and activity have been confirmed in studied marine hypoxic and anoxic environments (Fernandez et al., 2011; Hamersley et al., 2011; Jayakumar et al., 2012; Loescher et al., 2014). The study of non-cyanobacterial diazotrophic contribution to the ocean’s reactive nitrogen pool constitutes a new research paradigm of marine nitrogen cycling (Bombar et al., 2016). Breakthroughs in this field are indubitably instrumental to developing better understandings of the marine carbon cycle and its interactions with global change.

Although nitrogen fixation may be enhanced in hypoxic and suboxic seawater, loss of fixed nitrogen via denitrification and anammox is more eco-energetically favorable and thus nitrogen loss is the major microbial process in these environments (Lam and Kuypers, 2011). It has been estimated that oceanic OMZ seawater accounts for one third or more of fixed nitrogen loss on a global scale (Canfield et al., 2010b). Under hypoxic and anoxic conditions, enhanced availability of reduced inorganic chemicals such as ammonia/ammonium and sulfide as energy sources facilitates microbial carbon fixation that is coupled to nitrogen and sulfur cycling processes, including chemolithoautotrophic ammonia oxidation, nitrite oxidation, sulfur oxidation, and anammox. Some of these microbial processes are intensified particularly at or near the oxic-anoxic interfaces in the water columns (Füssel et al., 2012; Capone and Hutchins, 2013). These processes may help to restore the nitrogen balance by removal of excess nitrogen originated from riverine and terrestrial inputs in eutrophic estuarine and coastal waters. However, they may aggravate the scarcity of nitrogenous nutrients and exert further limitation on the ocean’s capacity of primary productivity and carbon sequestration in oceanic waters. A recent study has found that AOA and NOB in OMZs have high affinities for oxygen and nitrification (even at 5 nM O_2_) may control fixed nitrogen loss that is subsequently performed by denitrification and anammox (Bristow et al., 2016).

The scarcity of fixed nitrogen limits primary production and BP-mediated carbon sequestration in vast regions of the ocean (Moore et al., 2013). To make the situation even worse, the current fixed nitrogen pool of the ocean may be unbalanced and dwindling, caused by nitrogen loss via denitrification and anammox being significantly greater than nitrogen gain via nitrogen fixation (Codispoti et al., 2001; Mahaffey et al., 2005; Codispoti, 2007). Although uncertainties and debates remain about the conundrum of this imbalance (Gruber and Galloway, 2008; Canfield et al., 2010a; Zehr and Kudela, 2011; Großkopf et al., 2012; Voss et al., 2013; Devol, 2015; Klawonn et al., 2015b; Zhou et al., 2016), enhanced nitrogen loss by denitrification and anammox as a result of aggravated hypoxia, anoxia and acidification in coastal seas and oceanic OMZs may indeed diminish the marine fixed nitrogen reservoir under the impacts of increasing anthropogenic activities and global warming. This diminishment may constitute a positive feedback mechanism that speeds up global change by further limiting the ocean’s carbon sequestration capacity.

The marine hypoxic and anoxic environments are also the hotspots for the production of biogenic greenhouse gases such as N_2_O, CH_4_, and occasional H_2_S (Naqvi et al., 2010; Wright et al., 2012; Capone and Hutchins, 2013; Carolan et al., 2015; Murray et al., 2015; Kock et al., 2016). N_2_O is produced by many microbial processes and it is also the precursor of NO radicals that cause ozone destruction in the stratosphere (Carpenter et al., 2012; Schreiber et al., 2012; Voss et al., 2013; Mellbye et al., 2016). Hypoxic and anoxic environments that are rich in labile organic matter facilitate heterotrophic denitrification and the production of N_2_O as a metabolic intermediate (Gilly et al., 2013; Townsend-Small et al., 2014; Babbin et al., 2015; Castro-González and Farías, 2015). Autotrophic denitrification that couples denitrification with anaerobic chemolithoautotrophic sulfide oxidation also contributes to fixed nitrogen removal and N_2_O production in these environments (Shao et al., 2010; Ulloa et al., 2012). Anammox bacteria may produce N_2_O as well, via enzymatic NO detoxification (Kartal et al., 2007). N_2_O is also produced by AOA and AOB, though the detailed mechanisms involved in these two groups of aerobic microorganisms may be quite different (Kozlowski et al., 2016). Hypoxic conditions strongly stimulate AOB N_2_O production via the enzymatic nitrifier denitrification process, while N_2_O production in AOA may result from abiotic reactions (Codispoti, 2010; Zhu et al., 2013; Kozlowski et al., 2016).

Submicromolar O_2_ has been found to reversibly suppress anammox and denitrification, likely at the enzymatic level (Dalsgaard et al., 2014). Due to intracellular anammoxosomes, anammox bacteria may be more resistant to O_2_ suppression than denitrifying bacteria (Dalsgaard et al., 2014). Relative to anammox, denitrification is likely to be a more important N_2_O production process in hypoxic environments. It has also been found that sulfide, which accumulates in extremely anoxic environments or exists cryptically under hypoxic conditions (Canfield et al., 2010b; Glaubitz et al., 2013), strongly stimulates denitrifying N_2_O production without affecting the anammox process (Dalsgaard et al., 2014). Hypoxic and anoxic conditions are usually accompanied by environmental acidification, which may cause increased denitrifier N_2_O production (see above “Chemolithoautotrophic Responses to Ocean Acidification” section). Marine hypoxic and anoxic conditions influence the speciation and abundance of many trace elements and heavy metals, which may also influence the microbial production of greenhouse gasses via influencing the synthesis and activity of the involved enzymes (Glass and Orphan, 2012). Furthermore, many denitrifiers harbor truncated denitrifying pathways lacking the gene of N_2_O reductase for reducing N_2_O to N_2_, contributing to N_2_O production and accumulation in the environments (Jones et al., 2008; Richardson et al., 2009; Graf et al., 2014). Although shorter pathways of energy metabolism lowers ATP yield, they increase the ATP production rate and thus the maximal growth rate of the denitrifiers (Costa et al., 2006; Jones et al., 2008; Simon and Klotz, 2013), providing an eco-energetic advantage in environments rich in labile organic matter and nitrate.

The diverse microbial N_2_O production pathways and environmental controlling factors dictate the dynamics of the marine N_2_O reservoir. With the increasing expansion of both the oceanic OMZs and coastal hypoxic and anoxic water bodies, it is reasonable to predict that the microbial processes in these environments may contribute more to the production of N_2_O. This may constitute a positive feedback on global change. Currently, the relative contributions of the different microbial N_2_O production processes to the marine N_2_O reservoir and dynamics is still not reliably solved, particularly at the regional and global scales. This situation may be tackled by future investigations and modeling, in which the incorporation of the microbial eco-energetic constraints may be helpful.

### Chemolithoautotrophic Responses to Environmental Trace Element Variations and Heavy Metal Pollutions

Ecosystem energy flow interconnects with the interweaved biogeochemical cycles of carbon, nitrogen, phosphorus, sulfur, and many other elements such as biological trace metals (Falkowski et al., 2008; Jelen et al., 2016). Many of the microbial energetic processes are catalyzed by metalloenzymes (Nitschke et al., 2013). Although exergonic bioenergetic reactions are thermodynamically favorable, they are usually hindered kinetically by high activation barriers and need enzymes to speed up (Nitschke et al., 2013). Activities of metalloenzymes rely on various redox-active metal cofactors. Due to their plasticity in adopting different oxidation states and coordination environments in diverse enzyme molecules, redox-sensitive transition metals such as Fe, Ni, Cu, Zn, Co, Mo, W, V, and Mn are the key elements that constitute the metal cofactors in metalloenzymes (Klotz and Stein, 2008; Nitschke et al., 2013; Simon and Klotz, 2013; Gómez-Consarnau and Sañudo-Wilhelmy, 2015). The valence, speciation, solubility, adsorption, organic complexation and rates of redox processes of transition metals are subject to influences by physicochemical conditions such as environmental pH, O_2_ content and redox potentials (Byrne et al., 1988; Banks et al., 2012; Sunda, 2012; Scholz et al., 2014). The properties of transition metals in marine environments are influenced by diverse biological factors as well (Morel and Price, 2003; Gerringa et al., 2016). The recently proposed “Ferrojan Horse Hypothesis” highlights a newly discovered viral mechanism for the behaviors of Fe in marine environments (Bonnain et al., 2016). Under global change impacts such as those from ocean warming, acidification, and deoxygenation, the bioavailability and bioactivity of some of these transition metals may be altered (Hoffmann et al., 2012; Gledhill et al., 2015; Emerson, 2016; Stockdale et al., 2016). This may influence the synthesis and activity of certain key metalloenzymes that are involved in microbial energy metabolism, leading to changes of the composition and activity of the marine microbiota and further changes of the marine environment and its functions. For example, AOB employ the Fe-based electron transfer system for ammonia oxidation-mediated bioenergetic process, while AOA employ the Cu-based electron transfer system for ammonia oxidation-mediated energy metabolism (Walker et al., 2010; Santoro et al., 2015). Vast areas of marine environments are Fe-limited, particularly in the open oceans. The reliance on Cu other than Fe may provide AOA an eco-energetic advantage over AOB and contribute to the dominance of AOA in many marine environments (Walker et al., 2010). However, seawater Cu and Fe concentrations and speciation are subject to variation. The scarcity of bioavailable Cu in certain marine environments may impose a limitation on AOA abundance and activity (Jacquot et al., 2014; Shiozaki et al., 2016). There exist steep concentration gradients of Cu and Fe in marine OMZs, where suboxic and anoxic conditions decrease dissolved Cu concentrations but increase dissolved Fe concentrations and these trace metal profiles concur with the *in situ* Cu metalloenzyme gene profile of ammonia-oxidizing *Thaumarchaeota* and the Fe metalloenzyme gene profile of anammox *Planctomycetes*, respectively (Glass et al., 2015). The temporospatial distribution and dynamics of transition metals may be an important factor determining the temporospatial distribution and dynamics of the various microbial energetic pathways and functions in the ocean. Researches on this aspect are rare and certainly need to be strengthened.

Most of the microbial metalloenzymes are sensitive to the inhibitory effects of heavy metals. Genes encoding heavy metal resistance are much more abundant in the genome of *Ca.* Nitrososphaera gargensis isolated from a heavy metal-containing hot spring than in AOA isolated from marine environments (Spang et al., 2012). This indicates that AOA need specific genetic and biochemical mechanisms for heavy metal resistance and marine AOA may be sensitive to the inhibitory effects of heavy metals. Ongoing marine environmental changes caused by ocean warming, acidification, deoxygenation, eutrophication, and pollution may change the concentration, speciation, solubility and mobility of heavy metals as well, particularly in estuarine and coastal environments where heavy metal contaminations are usually prevalent (Atkinson et al., 2007; Millero et al., 2009; Gao et al., 2014; Zeng et al., 2015). The global change problem may be worsened by the compounding effects of heavy metals on the marine microbial processes and functions. The complex interactions between the energy metabolic processes and element biogeochemical cycles indicate that the eco-energetics of the marine microbiota need to be studied with a multidisciplinary effort (Klotz, 2010).

## Future Perspectives on Marine Chemolithoautotrophic Eco-Energetic Research

Microbial metabolism is driven by thermodynamic favorability, which is determined by the availability of free energy in the involved biochemical reactions. Energy is therefore an important constraint, along with nutrients, on the physiology of any organism and the structure and function of any ecosystem. For example, low light availability exerts an energy limitation on the photosynthetic productivity though nutrients are abundant in eutrophic estuaries with high seawater turbidity (Dang and Jiao, 2014). On the contrary, the lack of reduced inorganic chemicals such as NH_4_^+^/NH_3_ and H_2_S as energy substrates exerts a limitation on the chemolithoautotrophic primary production in the oxic deep oceans where nutrients such as nitrate and phosphate are usually available.

Environmental physicochemical conditions may exert important constraints on Gibbs energy yields and activity rates of the marine microbiota. For example, it has recently been found that the temperature-pH-salinity extremes exert a much stronger effect on the growth of anaerobically respiring and fermentative bacterial and archaeal strains than on the growth of aerobically respiring strains (Harrison et al., 2015). The difference in living parameter spaces between anaerobic microorganisms and aerobic microorganisms is likely related to their distinct eco-energetic properties. ATP yields of aerobically respiring microorganisms can be an order of magnitude higher than those of the anaerobically respiring or fermentative microorganisms, enabling better performances of aerobes in stress resistance, growth and activity over a broader range of physicochemical extremes (Harrison et al., 2015). This rule may be applicable to the chemolithoautotrophic microorganisms as well. The eco-energetic differences between the aerobic and anaerobic chemolithoautotrophic microorganisms may also affect their respective performances and functions under physicochemical extremes. Although it has not yet been systematically investigated, this hypothesis is important for understanding some of the mechanisms that lead to the compositional and functional shift of the marine microbiota under the various global change impacts.

Ecosystem energy flow involves diverse metabolic pathways (usually harbored by different microorganisms) and their interactions at various temporal and spatial scales. A community perspective is needed for the understanding and study of ocean biogeochemistry and eco-energetics (**Figure 1**) (Strom, 2008). Many ecophysiological activities and biogeochemical functions of the marine microbiota are carried out through microbial interactions including both cooperation and competition (Litchman et al., 2015; Dang and Lovell, 2016). For example, marine AOA usually have very small cell sizes and genomes (Martens-Habbena et al., 2009; Bayer et al., 2016), providing certain eco-energetic advantages particularly in oligotrophic environments (Batut et al., 2014; Martínez-Cano et al., 2015). Due to genome reduction, some marine AOA have lost the genes that encode for the catalase-peroxidase proteins (Kim et al., 2016). This energetic economy necessitates the dependency of these AOA on other co-occurring microorganisms for oxidative damage protection. The dependence of genome-reduced microorganisms on other microorganisms facilitates the development of metabolic collaborations and other mutualistic interactions in microbial communities. In addition to producing phosphonates for sharing with other microorganisms as a phosphorus source (Metcalf et al., 2012), marine AOA also harbor the genes for synthesis of vitamin B_12_ (Doxey et al., 2015), an essential cofactor required by many marine organisms (Gómez-Consarnau and Sañudo-Wilhelmy, 2015). Auxotrophy and physiological complementation, among many other microbial interactions, may help establish metabolic interconnectedness in natural microbial communities (Giovannoni et al., 2014; Garcia et al., 2015; Kouzuma et al., 2015). Microbial interactions are one of the key intrinsic properties of natural microbial communities that defines not only the composition and structure but also the activity and function of the communities (Hunt and Ward, 2015). Therefore, a systems ecology approach is needed for eco-energetic analyses of the marine ecosystems, in particular for a mechanistic understanding of the ecosystems’ driving force, the energy flow along the electron transfer pathways and redox exchange-induced matter fluxes within the marine microbiomes (Kolber, 2007).

In the past, investigations based on matter metabolism and fluxes became the mainstream of marine ecology research, though energy metabolism plays an equally important role on the structure and function of marine ecosystems. A few attempts have been made on eco-energetic analyses for certain marine environments (e.g., Amend et al., 2003; Akerman et al., 2011; Dahle et al., 2015; LaRowe and Amend, 2015; Bach, 2016). Most of these analyses focused on energy-limited environments such as the subseafloor deep biosphere where only the maintenance energy of the studied microbial community needs to be considered. However, community growth-related temporospatial variation is common in many marine environments, which need more sophisticated and dynamic eco-energetic processes to be taken into consideration for the analyses (Vallino and Algar, 2016). With the advance of the “omics” approaches such as metagenomics, metatranscriptomics, and metaproteomics for community metabolic network analyses, a comprehensive understanding of the mechanisms, processes and environmental responses may be obtained about the functions of marine microbial communities (Morris et al., 2010; Bodrossy, 2015; Reed et al., 2015; Perez-Garcia et al., 2016). These may also help to understand the genetic, biochemical and physiological constraints on the coupling or uncoupling of metabolic processes among different microorganisms or functional groups in an environment. Just like people need two legs to walk, the combination of the “omics” techniques with *in situ* energy and matter flux measurements or calculations may help to develop advanced biogeochemical models for better understanding and prediction of the processes and functions of the marine ecosystems and their responses to the global change impacts (Kolber, 2007; Soh and Hatzimanikatis, 2010; Vallino and Algar, 2016).

## Author Contributions

All authors listed have made a substantial, direct and intellectual contribution to the work, and approved it for publication.

## Conflict of Interest Statement

The authors declare that the research was conducted in the absence of any commercial or financial relationships that could be construed as a potential conflict of interest.

## Abbreviations

ANME: anaerobic methane oxidation
CBB: Calvin-Benson-Bassham cycle
rTCA: reductive tricarboxylic acid cycle
Comammox: complete oxidation of ammonia to nitrate
Anammox: anaerobic ammonium oxidation
DC/4-HB: dicarboxylate/4-hydroxybutyrate cycle
WL: Wood-Ljungdahl pathway (i.e., reductive acetyl-CoA pathway)
3-HP/4-HB: 3-hydroxypropionate/4-hydroxybutyrate cycle
